# Match and Mismatch between Lived Experiences of Daytime Sleepiness and Diagnostic Instruments: A Qualitative Study amongst Patients with Sleep Disorders

**DOI:** 10.3390/clockssleep6010003

**Published:** 2024-01-05

**Authors:** Vaida T. R. Verhoef, Karin C. H. J. Smolders, Lysanne Remmelswaal, Geert Peeters, Sebastiaan Overeem, Yvonne A. W. de Kort

**Affiliations:** 1 Human-Technology Interaction, Department of Industrial Engineering and Innovation Sciences, Eindhoven University of Technology, P.O. Box 513, 5600 MB Eindhoven, The Netherlands; k.c.h.j.smolders@tue.nl (K.C.H.J.S.);; 2 Center for Sleep Medicine Kempenhaeghe, 5591 VE Heeze, The Netherlands; peetersg@kempenhaeghe.nl (G.P.); s.overeem@tue.nl (S.O.); 3 Department of Electrical Engineering, Eindhoven University of Technology, 5612 AP Eindhoven, The Netherlands

**Keywords:** daytime sleepiness, residual complaints, fatigue, tiredness, obstructive sleep apnea, narcolepsy

## Abstract

Excessive daytime sleepiness is a common symptom of sleep disorders. Despite its prevalence, it remains difficult to define, detect, and address. The difficulties surrounding sleepiness have been linked to an ambiguous conceptualization, a large variety of scales and measures, and the overlap with other constructs, such as fatigue. The present study aims to investigate patients’ descriptions of sleepiness-related daytime complaints and their phenomenology. We performed semi-directed interviews with patients diagnosed with obstructive sleep apnea (N = 15) or narcolepsy (N = 5). The interviewers took care of utilizing the participants’ terminology when describing daytime complaints related to their sleep disorder. Various aspects of the daytime complaints were investigated, such as their description and temporality. The transcribed content was thematically analyzed using an eclectic coding system, yielding five themes. The participants used different interchangeable descriptors (tired, sleepy, fatigued, exhausted) to express their daytime complaints. They enriched their description with indexes of magnitude (ranging from ‘not especially’ to ‘most gigantic, extreme’), oppositions to other states (using antipodes like energy, alertness, wakefulness, or rest), and indications of fluctuations over the day. Interestingly, the participants often used metaphors to express their experiences and their struggles. The lived experiences of the patients were found to not always align with common self-reported monitoring tools of sleepiness and to relate only in part with current conceptions. In practice, it is important to probe daytime complaints, such as daytime sleepiness, with a broader consideration, for example, by exploring antipodes, consequences, and time-of-day fluctuations.

## 1. Introduction

Excessive daytime sleepiness (EDS) is a common symptom across various sleep disorders. It may be a primary symptom, such as in narcolepsy, or a consequence of nocturnal dysfunction, such as in obstructive sleep apnea (OSA) [1,2,3,4], where EDS may still last after treatment [5]. Deemed a “challenge for the practicing neurologist” by Guilleminault and Brooks in 2001 [6], daytime sleepiness remains a puzzle as, despite its relevance, it is often unrecognized or unaddressed [7,8,9]. The clinical complexity of EDS has been linked, among others, to the ambiguous conceptualization of sleepiness and the overlap with other constructs [9] like hypersomnia, hypersomnolence [10], drowsiness [11], sleep inertia, fatigue [8,12,13,14], or tiredness [15]. Many have defined daytime sleepiness as the propensity to fall asleep during the day, sometimes in inappropriate contexts [16], much like in narcolepsy or idiopathic hypersomnia. Other definitions also encapsulate perceived sleepiness and the consequences of daytime sleepiness [11,17,18,19]. This conceptualization relates partly to the definition of hypersomnolence proposed in recent reviews [11,18,19]: a disabling symptom which includes EDS, an excessive need for sleep, and disturbances upon awakening. According to their model, EDS is sub-defined by three categories of symptoms, as follows: continuous non-imposing drowsiness, sleep propensity with voluntary or involuntary bouts of sleep, and automatic behaviors [20]. While this conceptualization unites various dimensions of daytime sleepiness, it does not necessarily align well with common diagnostical metrics. 

Cost-effective and user-friendly, self-reported assessments of hypersomnolence and, specifically, of EDS are the most common tools both in clinical practice and in research. However, they are not optimal. In fact, the scores of patients who experience daytime sleepiness can appear either low (due to, e.g., compensatory behaviors or a lack of relevance in the scales vis à vis patients’ experiences) or high but inconsistent with clinicians’ concept of EDS or objective measures. Studies have suggested that different subjective scales [21] do not necessarily correlate nor do they always correlate with objective measures [17,22,23]. The shortcomings of monitoring are also highlighted by researchers who question the relevance, applications, and influencing factors of common scales such as the Epworth Sleepiness Scale (ESS) [24]. The lack of concurrence between metrics might stem from the multidimensional nature of sleepiness [17,24,25,26]. Perhaps, it also stems from a mismatch between the current conceptualization of daytime sleepiness, the terminologies in common subjective measures, and patients’ actual experiences. 

In order to further advance our understanding of daytime sleepiness [11,18,19], we argue that a bottom–up approach could be helpful. Through the perspective of patients, we can evaluate to what extent patients’ experiences and descriptions of their complaints align with current conceptualizations of sleepiness and with common subjective scales. We, thus, attempt (1) to obtain a better grasp of the extent and nature of the symptoms that patients experience and (2) to understand the vocabulary they use as first-hand owners of these experiences, in order to then (3) reflect on the fittingness and completeness of diagnostic tools. The current study takes a qualitative approach to investigate daytime complaints, with a specific focus on daytime sleepiness, among patients diagnosed with a sleep disorder as the focal phenomenon. Clarifying daytime symptoms as voiced and phrased by patients with sleep disorders could be a first step towards the improvement of subjective diagnostic instruments. Furthermore, gaining knowledge regarding the temporal dynamics of daytime symptoms can be useful for determining an optimal measurement resolution for the assessment of sleepiness-related symptoms, as well as for the temporal tuning of future interventions.

## 2. Results

### 2.1. General Description

The interviews ranged from 27.5 to almost 53 min long, with an average of 37 min and 30 s. Aiming to include participants affected with potentially different dimensions of daytime sleepiness, we recruited those diagnosed with narcolepsy and OSA. In total, 1923 segments were coded across both diagnostic groups, with 1498 segments corresponding to the participants diagnosed with sleep apnea and 425 segments related to the participants with a diagnosis of narcolepsy. Most of the segments pertained to the description of daytime complaints related to the patients’ disorders, after which questions on bad and good days or the day prior to the interview offered the most content. 

From the analysis of the data with a focus on how the patients described the daytime consequences of their sleep disorders, five key findings emerged as themes: distinct and yet closely related descriptors of daytime complaints (Theme 1), the use of antipodes to help describe (Theme 2), an image or a metaphor to illustrate (Theme 3), other physical and mental signs to help describe (Theme 4), and a time space for daytime complaints related to sleepiness, tiredness, or fatigue (Theme 5). 

In the description of the themes below, each citation is linked with a specific participant in a code, for example N1F21. In this instance, the first letter refers to the participant as someone with a diagnosis of narcolepsy (N), with participant number (1), of feminine gender (F), and twenty-one years old (21). 

### 2.2. Theme 1: Distinct and Yet Closely Related Descriptors of Core Daytime Symptoms

When asked about possible daytime experiences related to their sleep disorder, all the participants referred to tiredness, sleepiness, and/or fatigue. The corpus acquired through the interviews depicts a myriad of terms of which many were used simultaneously. These terms and states varied between tiredness, fatigue, exhaustion, unrest, sleepiness, sleep attacks, sleep deprivation, and unwellness. 

#### 2.2.1. Tired

Almost all the participants expressed being tired, which appears to be the most commonly used term throughout the interviews. To illustrate many other statements, participant A1M56 referred to his state as “*you are tired, you just don’t get started*”. 

#### 2.2.2. Fatigued

Interestingly, in our participants, fatigue was an experience only referred to by the participants diagnosed with sleep apnea. Although the terms fatigue or fatigued were used verbatim (for example, “*really pure, really that fatigue*”, patient A15F65), the descriptions of the participants were often related to either another state (like sleepiness) or to somatic experiences. To illustrate, participant A7M52 mentioned being “*so fatigued that when I touch my pillow, I sleep*”. 

#### 2.2.3. Exhausted

The second most common descriptor was exhaustion, either verbatim or through synonyms. For participant A3F55, this experience translated to expressing “*I’m always dead and dead tired*” during her description of her tiredness on the day prior to the interview. Similarly, participant A4F73 described her experience as “*kind of exhausted*” and then “*dead tired*”. In fact, for some, like participant A9M49, little mundane tasks like cooking seemed only possible “*really on my last legs, so to speak, on my last strength*”. It seems that exhaustion concerns mostly sleep apnea patients. However, one participant with narcolepsy (N1F21) also indicated exhaustion as a primary daytime concern. 

#### 2.2.4. Sleepy

When it comes to feelings of sleepiness, interestingly, only seldomly was the word sleepy used. However, sleepiness was described by both diagnostical groups as the tendency to fall asleep in certain situations. For the participants with narcolepsy, this was reflected in falling asleep in various calm situations, just like participant N1F21 stated “*as soon as I read a page of something, I fall asleep*”. The ease of falling asleep like participant N5F53 mentioned (“*I fell asleep easily during the day*”) can also be found among the experiences of the participants with sleep apnea. Participant A11F62 used the example of falling asleep as a passenger in a car (“*when my husband was driving, we weren’t even 10 min on the highway that I was asleep*”); others used examples of falling asleep while sitting down or watching TV (“*But right now I sometimes manage to fall asleep in my chair*”, participant A3F55). Very few participants described their experiences as sleep attack or sleep pressure.

#### 2.2.5. Interchangeable Descriptors

The corpus acquired through the interviews depicts a myriad of terms of which many seemed to be used interchangeably. Some participants did make an explicit distinction between tiredness and sleepiness (“*I was not sleepy. I was tired!*”, participant A10F52, and “*I actually never feel tired, I feel sleepy, but I’m not tired*”, participant N5F53). Moreover, participant N4M22 also described his experience in a distinctive manner by saying “*I’m feeling tired, but it’s usually not from the “you want to sleep” type so to speak*”. Yet, later in the interview, participant N4M22 specified his experience as “*you just feel really tired, sleepy*”. Similarly, some participants associated their tiredness with other experiences like the need to sleep (“*so tired that I almost fell asleep*”, participant A6F66), a state that could be linked to feelings of sleepiness, or the feeling of exhaustion (“*I’m tired. I’m dead tired*” or “*and not just tired, but really exhausted*”, participant N1F21). Hence, the use of multiple terms by all the participants blurs the distinctions.

#### 2.2.6. Magnitude

In conjunction with descriptors, the participants used another method to describe and often emphasize their struggles—levels of magnitude. Indeed, the participants were not only feeling tired but either “*not especially tired*” (participant A13F74), “*just tired*” (participant N1F21), or “*really tired*” (participant A6F66), “*so tired*” (participant A1M56), “*very tired*” (participant A2F54), or even “*extra tired*” and “*really such a gigantic intense tiredness*” (participant A6F66). When it comes to fatigue, similar levels could be found, if not with a smaller range: “*really very fatigued*” (participant A7M52), “*extra fatigued*” (participant A9M49), and “*the most extreme fatigue*” (participant A10F52). Even exhaustion was emphasized (“*very exhausted*”, participant A4F73), while sleepiness did not appear to be expressed in the same way (i.e., with adjectives to detail the magnitude of the experience). The use of levelling words differed not only between interviews but also throughout some of them, as an indication of severity. 

### 2.3. Theme 2: The Use of Antipodes to Help Describe Daytime Complaints

The use of antipodes was popular among the participants to enhance or complete their descriptions. We define antipodes as terms used by the participants in a comparison/opposition to their daytime complaints. For example, when expressing tiredness or sleepiness, the participants also defined their experience as a lack of energy or a lack of alertness. In this instance, energy and alertness are antipodes of tiredness and/or sleepiness. 

#### 2.3.1. Energy, Alertness, Rest, Wakefulness, and Efficiency: All Antipodes

The participants across both diagnostic groups often presented their struggle as a lack of energy. For example, participant A15F65 stated “*Just zero energy, done*”. Another telling example was the following description of tiredness by participant A11F62: “*just run out of energy*”. When asked to describe a good day in comparison to a bad day, most of the participants characterized the distinction using the term energy. A good day could be one during which one “*jump[s] on [the] bike*” or is “*energetic*” (A2F54) or one during which one “*can start the day full of energy, [and] can do all the things [one] needs to do*” (A14M61). 

The regular absence of energy was, however, not a consensus among all the participants; participant N5F53 even expressed that “*it is never like I don’t have energy or something*”. And some participants, like patient N5F53, preferred to relate their struggle to, for example, something on a continuum from sleepiness (“*I feel sleepy*”) to alertness: “*Yeah, that, you know, that I don’t feel alert*”. Others described a good day as one “*when I’m clear, then I’m faster*” (A8F55), “*when I’m also more alert, […] I function better*” (N3M20), or when, with the help of physical activity and good weather, “*the clarity in my head is at such moments very good*” (A15F65). 

While less used by the participants, concepts of rest, wakefulness, or the capacity to carry out tasks were also mentioned as antipodes to feeling tired, sleepy, or fatigued. Participant A10F52 described her struggle as “*I’m not really rested*”, echoing the experiences of some other participants with sleep apnea. On a similar path, another participant related the efficiency of a nap to feeling “*much more rested, so to say*” (N4M22). A telling example of wakefulness came from participant A10F52, who first highlighted a struggle in “*feeling not awake*” on a regular morning and then described a good day by being “*awake awake*”. 

#### 2.3.2. A Mirror to Descriptors: Interchangeable Antipodes

As described above, the participants did not always restrict the description of their experiences to one term. For example, participant N3M20 mentioned “*it cost me a lot of energy to stay alert, to stay effective*” in tiredness-enhancing weather conditions and then separated alertness from wakefulness later in his interview by saying “*then I notice a difference in alertness and the way of being awake, so to speak*”. When it comes to the antipode–descriptor combination, it appears that no pair can be separated and defined. At a first glance, some of the participants’ descriptions seemed to clearly outline pairs such as energy–fatigue (“*then the fatigue disappeared and the energy came back much more*”, participant A4F73) or sleepiness–alertness (“*the sleepiness, and that my head is out of focus*”, participant N5F53). However, if one looks at the ensemble of the interviews, the pairs seem to dissolve into the mix of descriptors. 

### 2.4. Theme 3: An Image or a Metaphor to Illustrate Daytime Symptoms

Recurrent metaphors were used to contribute to the description of daytime concerns. 

#### 2.4.1. A Reservoir Going on Empty

Most predominately, it seems that the patients viewed their experiences as an “emptying reservoir” of perhaps “energy” or force, calling it either a “tank” or a “battery”. To illustrate, participant A10F52 described her experience as “*you don’t have any reserves, you are totally inflexible*”, just like participant A11F62 mentioned that “*it is as if some kind of tank is empty*” or like participant A9M49 (“*my tank is actually completely empty around 11–11:30*”). On a similar level, participant A14M61 explained the effect of taking a break (“*as if you have recharged your battery for a while*”), just like taking a nap would give “*a sort of very small, small power boost*” to participant N1F21.

#### 2.4.2. A Threshold Not to Cross

Another metaphor was linked with the idea of an imaginary, sometimes inevitable “threshold” up until which a patient can handle their tiredness, sleepiness, or fatigue. Among others, participant A12M69 considered that “*the irritability and fatigue remain reasonably within the limits, but I do feel I’m too close to those limits throughout the day*”. For others, it seemed that “*the whole buffer was gone*” (participant A10F52) […], thus needing to adapt their lives, as participant A7M52 explained: “*I also have to learn to deal with that and not to push my limits again, so to speak, so I just have to learn to live very carefully*”. 

#### 2.4.3. A Cost and a Bank Account Close to Zero

Moreover, a link was drawn with the analogy of “costs and bank accounts”, where a delicate balance between natural costs and steep deficits was at play in how the patients maneuvered their symptoms in their daily life. To illustrate, participant A5M50 explained his perception of his exhaustion and tiredness as “*the phenomenon of a savings account and to be in overdraft. If you go on spending long enough, yes, at some point you just hit the limit and then everything just stops. Then your debit card no longer works, and it does for me too*”. 

#### 2.4.4. A Mental Fog

Analogies and metaphors were also used in relation to the more physical, perceivable aspects of the symptoms. Some patients expressed their experiences as a clouding of consciousness in their head, a sort of mental fog. In particular, participant A8F55 described a “*thick mist in my head*” as a result of tiredness, just like participant A1M56 described his fatigue as “*a kind of brain fog*”, and participant N5F53 described the following: “*Sleepiness is kind of […] that it all becomes a kind of cotton wool in my head, that I can’t think clearly anymore, that I’m not sharp anymore*”.

#### 2.4.5. A Slowdown

Another metaphor that related to the consequence of their symptoms (be it tiredness, sleepiness, exhaustion, or fatigue), was the “slowdown” both in how the body moved and how cognition was affected. A salient example is the notion of “walking through syrup” (participant A2F54). More explicitly, participant A8F55 defined her experience by saying “*I’m slow, really slow. Even physically I am slower*”.

The metaphors of cost and reservoir were the most popular, used by most of the participants affected by sleep apnea or narcolepsy. Other images, like inertia or the cloudy brain, were scarcer and only used by the participants with a diagnosis of sleep apnea. […]

### 2.5. Theme 4: Physical or Mental Signs to Help Describe Daytime Experiences

The repercussions of tiredness, sleepiness, or fatigue were numerous and ranged in nature from somatic, cognitive, affective, and behavioral to social. 

#### 2.5.1. Physical Consequences

Many of the participants’ descriptions included physical consequences of the tiredness, sleepiness, or fatigue they had been experiencing. Most of the participants described drooping eyes, like participant A1M56 described alongside his feelings of fatigue: “*your eyes close, the shutters close, whether [you] like it or not*”. This common phenomenon can be linked to a feeling of heaviness that seems to take over the body: the body “*feels heavy*” (participant A8F55), and, apart from the eyes, the “*face is hanging somewhere on [the] knees*” (participant N1F21), “*arms, legs… were incredibly heavy*” (participant A10F52), with subsequent effects such as “*the movement starts to get heavier*” (participant N3M20). A few of the participants mentioned the sensation of “*yawning a lot*” (A10F52). For some participants, this physical component was similar to feeling unwell, “*becoming nauseous*” as a consequence of fatigue (participant A7M52), or experiencing a “*clammy feeling*” (participant A13F74), with sometimes the addition of pain, like participant A8F55 said: “*That was very physical. I became nauseous, I had pain in my back, pain in my stomach*”. For other participants, the unwellness came from the feeling of dizziness, either alongside yawning and lack of concentration, or alongside a lightness in the head (participant A3F55), or after intense sudden movement (“*And then you bend down, get up, and you’re actually a little dizzy*”, participant A14M61). While participant A9M49 added to his description by simply saying “I feel so lousy”, others were more descriptive, like participant A3F55 (“*I completely collapsed again and felt so sick*”) or participant A14M61 (“*It’s like you’ve been drinking, that you’re drunk*”). For a small subset of participants, the physical symptoms and bodily sensations or even tiredness itself were completed with the experience of “hunger” (participant A4F73). Participant A11F62, for instance, mentioned “*I know I’m tired, so I’m going to eat*” as a direct consequence to her daytime experience. That experience can then result, for example, in “*snacking, […] look[ing] for energy-rich products that contain a lot of sugars*” (participant A6F66). 

While the heaviness and the sensation around the eyes were seen in the experiences of both diagnostical groups, other physical sensations presented above were only recalled by the participants with a diagnosis of sleep apnea. In some quotes, the link between a certain descriptor and a physical one can be drawn. However […], when one looks at the corpus of information, such links seem to dissolve between the various descriptors. 

#### 2.5.2. Cognitive Consequences

Apart from these somatic representations, the participants often referred to the cognitive consequences of their struggles. Almost all the participants mentioned suffering from a lack of concentration. For participant A5M50, there was “*no more concentration*”, “*I can’t think clearly anymore*”. This sentiment seemed to be shared by many of the participants of both diagnostic groups and was sometimes directly linked with tiredness (e.g., “*so the concentration is then linked to that, to the tiredness*”, participant N4M22). For some participants, this lack of concentration was associated with atypical behaviors or impressions, like “*put[ting] plates in the washing machine and the laundry in the dishwasher*” (participant A5M50), “*hear[ing] people from afar*” (participant A3F55) during a close conversation, or not remembering a conversation in which one had participated (“*all kind of things can be said to me, but I don’t remember that afterwards*”, participant 12M69). Less common cognitive struggles related to memory and language, reflected either in “*forgetting simple things*” (participant A5M50), or the tip-of-the-tongue phenomena, described by participant A7M52 as follows: “*You are so tired that you start stuttering a bit, but then you are really exhausted that you can no longer come up with words, that you no longer remember names while you know the name of someone very well. Only at that moment you just don’t get there anymore*”. 

#### 2.5.3. Affective Consequences

It is difficult to disentangle affective effects from any potential comorbid psychopathology occurring in parallel to the sleep disorder. Nevertheless, some common affective consequences were mentioned. When asked about more details on her tiredness, participant A2F54 mentioned a lack of motivation, even for very mundane tasks: “*things we see and want, […], but you quickly think: yes that is way too much work*”. Even more than a diminished drive, participant A3F55 described it as “*despondency*”: “*So that’s what you get from tiredness, […] a kind of lameness*… *that you think: well never mind it all, you know?*”. The participants also related other emotional symptoms to their experiences, such as an increase in emotional sensibility. For example, participant N5F53 might suddenly “*get emotional in the afternoon*”, or participant A8F55 expressed the following: “*when I was really dead tired, I also just sobbed faster*”. The despondency and emotional susceptibility can be linked to other negative thoughts and affect sometimes found in both diagnostical groups; the participants mentioned feeling “*very depressed*” (participant A3F55) as a result of their sleep disorder or feeling “*so somber*” (participant N1F21). 

The participants in both groups mentioned experiencing “*irritability*” (participant A10F52) as a response to their diagnosis and as a sign of their state. Approximately half of the participants diagnosed with sleep apnea and one participant with narcolepsy mentioned “*a very high irritation level*” (participant A6F66), also expressed as a “*short fuse*” (participant A8F55). A salient example comes from participant A14M61: “*I have a pretty short fuse when I’m tired*”. This irritability was sometimes associated with social consequences. For participant A6F66, short fuses and other consequences end up making her “*an unpleasant person*”, while participant A14M61 reports “*grumbl[ing] quite a bit about everything and everyone*” or “*drink[ing] everyone’s blood so to speak*” (participant A3F55). For other participants, without any links to irritability, “*social contact is just difficult, because of course you have to put energy into it, and of course you don’t have that*” (participant N5F53), a sentiment which was voiced in both diagnostical groups. 

#### 2.5.4. Mental vs. Physical Consequences

In the ensemble of the participants’ experiences, it seems important to note a distinction between mental and physical experiences. A good example comes from the experience of participant A10F52: “*My body still wants to sleep and my head is grinding, something like that*”. Indeed, whether through the use of metaphors or related symptoms, the participants sometimes highlighted this contrast. Participant N4M22 explained feeling “*physically tired in your muscles, but you also really feel like sleepy tired*”. The use of the term “*physically tired*” was found across various descriptions and in both groups. Participant N4M22′s description comes close to that of participant A6F66, i.e., “*so it indeed was both physical and mental I think*”. For some, it seemed that the complaint was “*mainly mental*” (participant N3M20), while for other participants the symptoms were “*mainly physical*” (participant A6F66). 

### 2.6. Theme 5: A Time Space for the Daytime Complaints

Unprompted, the participants used different adjectives to express the timing or duration of their experiences. Interestingly, almost all the participants described their struggles as a constant burden. Indeed, descriptors of tiredness, sleepiness, or fatigue were often accompanied by an adjective like “*always*”, “*still*”, “*chronic*”, and “*constant*” or verbs like “*stay*” or “*continue*”. To illustrate, participant N1F21 mentioned “*being tired, that stays, day after day, every second of the day*”. As such, this agrees with the idea of some participants that their sleep disorders have “*consequences for the rest of your life*” (A14M61). 

However, when prompted by questions about the timescale of their daytime struggles, the participants did specify variations in intensity. In general, the participants affected by either sleep apnea or narcolepsy experienced tiredness, sleepiness, or fatigue as soon as they woke up in the morning. Like many, participant N2F47 “*wakes up really tired in the morning*”, just as participant A3F55′s experience (“*it is the worst when you get up in the morning*”). Only a couple of participants (all affected by sleep apnea) mentioned feeling like participant A10F52: “*I’m at my best in the morning*”. When the afternoon comes, quite often these participants experienced a “*dip around, I think, 3 pm*” (A8F55), sometimes earlier or later. Participant A10F52 felt well in the morning and then described her daytime complaints as follows: “*in the afternoon, those are the worst*”. But, the dip can also be found among participants who experienced daytime complaints in the morning. When it comes to the evening, the experiences of the participants can be distinguished into either improved or worsened, independently from prior variations throughout the day. For some participants, like participant A2F54, “*in the evening, [it] often goes better*”, whilst others, like participant A8F55, felt “*at the end of day… exhausted*”. 

When taken together across participants, time-of-day dependencies could indicate either an improvement or a worsening of daytime complaints throughout the day. However, when prompted by questions, only very few participants mentioned feeling “it coming” (N5F53). Instead, most of the participants described their experiences like participant A10F52: “*It really happens to me quite suddenly*” or “*in one go, bam!*” (A14M61). 

Although some “*kind of day pattern*” (A1M56) can be recognized in every participant, no clear distinction can be made between the diagnostical groups. 

## 3. Discussion

We investigated the unbiased terminology used by patients diagnosed with OSA and narcolepsy when describing their daytime complaints related to sleepiness. We performed this to grasp the magnitude, range, and nature of the complaints but also to evaluate to what extent they relate to common diagnostic tools. 

### 3.1. Conceptualization of Sleepiness Amongst Daytime Complaints 

Although sleepiness has been established as a core symptom of OSA and narcolepsy, terms specifically describing this daytime complaint were only scarcely used in the present corpus. In fact, the participants mentioned tiredness, fatigue, and exhaustion more frequently than sleepiness. The use of different descriptors was highlighted on an interindividual and on an intraindividual level and paralleled the variation observed in the mixed use of antipodes of daytime complaints. 

First and foremost, daytime sleepiness is not only related to the act of (involuntarily) falling asleep during the day. In fact, the participants in this study, while under treatment, rarely mentioned impromptu sleep episodes. However, they remained affected by somatic sensations associated with sleep pressure (heavy limbs, drooping eyelids, etc.) or by the effect that sleepiness (or other related symptoms) could have on their mood and cognitive abilities. In this sense, the residual complaints reported in this study extend beyond the conception of EDS by Gandhi and collaborators, for whom EDS equals involuntarily falling asleep during the day, prolonged diurnal sleep periods, and/or sleep inertia [27]. We saw similarities with the consequences of sleep inertia (namely irritability and automatic behavior) as introduced by Gandhi et al. [27]. However, we mostly noted parallels with the sleepiness dimensions introduced by Kim and Young [17] or with the conception and overview of hypersomnolence offered by Lopez et al. [11] and Martin et al. [18,19]. According to Lopez et al. and Martin et al., EDS is not only related to past or impending sleep episodes but also includes sensations and behavioral disturbances throughout the day. Taken together, our participants experienced a complex network of daytime complaints, with links to perturbations upon awakening and other aspects or repercussions of hypersomnolence [11,18,19,27]. This network is illustrated by the wide array of descriptors and consequences described by our participants. 

The diversity of interchangeable terms for potentially related symptoms complicates the evaluation of daytime complaints. If we go back to its definition, fatigue is a multidimensional state often mentioned as a persistent lack of energy with various consequences on mood, behavior, and physiology [28,29]. This definition echoes multiple observations in our results, notably the opposition between a descriptor (fatigue) with an antipode (energy), the various consequences of the daytime complaints, but also the continuity of the symptom. However, these characteristics can also be traced back to other descriptors used by our participants: tiredness, sleepiness, or exhaustion. 

In line with our findings, a study performed amongst a sample of OSA patients showed that the patients used the terms “fatigue”, “tiredness”, or “a lack of energy” more often than they reported sleepiness [15]. Combining this with the knowledge that the terms “fatigue”, “sleepiness”, and “tiredness” are sometimes used as synonyms for each other [26] suggests that “sleepiness” is not the terminology used consistently by patients to describe their daytime symptoms. While comparing OSA patients with non-OSA patients referred to a sleep clinic, Sunwoo et al. found no significant differences in ESS scores, but these groups did show significant differences in their responses to items of an in-lab developed scale named “Language of sleepiness”, which echoed our participants’ descriptions such as, for example, “I lack energy”, “I am tired all the time”, “I am fatigued”, and “I am often drowsy” [30]. The non-OSA patients scored higher on these items. Yet, when the authors compared the OSA patients classified to be with EDS (ESS ≥ 10) or without EDS, complaints probed with the language of sleepiness scale were more severe in the patients with EDS. These results highlight not only the overlap between sleepiness and fatigue but also the importance of considering daytime complaints beyond sleepiness among patients diagnosed with sleep disorders and individuals who experience sleep disruption without a clear diagnosis. 

Perhaps the terms exhaustion, tiredness, fatigue, and sleepiness belong to a common continuum, only varying in their severity. In fact, a recent semantic investigation performed among young healthy participants found significant differences between the terms “sleepy”, “drowsy”, “tired”, and “fatigued” with an increasing negative valence across those four words [31] and significant distinctions in potency between “fatigued” and either “sleepy” or “tired”. However, no significant differences in potency were found between “sleepy”, “tired”, and “drowsy”, suggesting that these adjectives carry similar weight or strength. While relevant, Long et al. [31] did not take “exhaustion” into account and their observations among young healthy participants might differ from the lived experiences of patients. 

### 3.2. Assessing Daytime Sleepiness

The terminology behind daytime complaints used by patients is important to consider when employing self-report scales in clinical practice and in research. As more than 50% of the tools used to measure daytime sleepiness are self-reports [18], it seems highly relevant to assess the similarities and differences between common scales and patients’ experiences. In fact, a clear mapping between questionnaire items and patients’ lived experiences is crucial for accurately monitoring and quantifying patients’ daytime complaints. Throughout the variety of terms used by the participants, some aspects call back on the various scales measuring daytime sleepiness, fatigue, or exhaustion. For instance, the participants sometimes described their struggles as falling asleep in various common situations, much like stated in the Epworth Sleepiness Scale (ESS) [32]. Yet, according to the participants’ own terms, these struggles were not only related to sleepiness but also to tiredness, exhaustion, or fatigue, which then questions the specificity of the scale items toward sleepiness. 

The participants’ use of antipodes and magnitude matches the Karolinska Sleepiness Scale (KSS) [33], which opposes alertness to sleepiness through different levels. However, we also noted the use of other antipode–daytime complaint combinations, such as energy–tiredness or rest–tiredness. As our participants described (cognitive) consequences on their daily lives, we note a parallelism with the Daytime Sleepiness Perception Scales (DSPS-4) [34]. Developed as a short (four-item) questionnaire measuring trait sleepiness among insomnia patients, the DSPS-4 asks to rate the frequency (*Never* to *Always*) of items assessing the need for sleep, the impact of sleepiness on performance, and sleepiness as a hindrance to daytime functioning. However, while the scale does align with some of our participants’ experiences, it uses terms like *sleepiness* or *sleepy* exclusively, which, according to our findings, might not be the terms typically used by patients. 

While the Stanford Sleepiness Scale (SSS) [35] offers a wider variety of descriptors in a spectrum going from feelings of vitality to impending sleep onset, the labels seem also distanced from our data due to the richness of the interchangeable descriptors and the levels of magnitude used by virtually all the participants. If the participants used levels to detail their struggles, those were used to indicate the severity of an already existing daytime complaint instead of offering an equilibrated opposition between the complaint and its opposite, as can be found in both the KSS and the SSS. In other words, only part of the scales would be relatable to the participants in this study. With the data presented here, an argument can be made that patients are already very affected by sleepiness or fatigue at a baseline level, and, as such, the existing scales might not be sufficiently sensitive or able to capture a worsening when it comes to monitoring said conditions. In addition, the scales might not echo the experience of patients simply by using a deviating terminology. In fact, the most common scales for EDS use drowsiness, sleepiness, concentration, and the opposition to alertness, whereas fatigue, tiredness, or energy are hardly used. As a result, these scales may miss an important aspect of EDS or a disabling aspect of the daytime complaints, hindering following treatment or guidance. We can also hypothesize that the treatment of sleepiness, although effectively alleviating involuntary diurnal sleep episodes or an excessive need for sleep, may not address the other aspects related to EDS or fatigue. The scales commonly used could then be less sensitive to those (sometimes severe) residual complaints. 

Contrary to the scales commonly used to probe daytime sleepiness, questionnaires used to measure fatigue, like the Chalder Fatigue Scale (CFS) [36], the Fatigue Assessment Scale (FAS) [37], and the checklist individual strength (CIS20R) [29], seem to relate more closely to the findings of the present study. These scales investigate levels of tiredness, sleepiness, and/or exhaustion while also measuring levels of energy or fitness, reflecting the diversity of descriptors used by our participants as well as the use of antipodes. Both the above-mentioned scales take into consideration physical symptoms and cognitive difficulties associated with the complaints. Other items report on aspects that can be linked to motivation or despondency, also mentioned by some of our participants. In a similar vein, a scale used to monitor levels of exhaustion (Karolinska Exhaustion Scale), often used amongst people affected by burnout, also utilizes items that mirror the lived experiences of our participants [38]. The scale is divided into four sections covering the absence of recovery (items like “Being tired all the time”), cognitive repercussions (e.g., “Difficulties with concentrating on a longer text, news article or book”), somatic representations (e.g., “Muscle weakness or numbness in arms or legs”), and emotional consequences (e.g., feeling “More irritated or angry”).

Among narcoleptic patients, fatigue and sleepiness have been found to be distinguishable and separate yet sometimes coexisting constructs [39], which would explain the use of various descriptors. Yet, in our group, narcoleptic patients preferred to describe their struggles as tiredness and exhaustion more than as sleepiness or fatigue. When it comes to our OSA patients, questions can be raised regarding whether they do experience EDS. However, our observations are strikingly similar to those by Waldman and colleagues [40] among OSA patients diagnosed with EDS. Just like their participants, our participants reported a preferential use of the term tiredness. Other descriptions like “brain fog”, exhaustion, and fatigue were also outlined in the current study, much like the consequences sorted by Waldman and colleagues [40] in their framework regarding EDS patients’ health-related quality of life (HRQOL). Our study deviated from this study in that we used interviews instead of focus groups to avoid group-related bias. Nonetheless, we find important similarities between studies, which raise important questions on the nature of EDS, such as the following: should it be dissociated from fatigue or exhaustion, or is it an ensemble of related symptoms? 

Another important aspect we encountered that remains missing from common sleepiness and fatigue measurement protocols pertains to intradaily variations in the symptoms. Most scales probe an average over a period of time (e.g., the last month). Even when sleepiness—the only state amongst tiredness and fatigue which can be assessed momentarily through a validated scale (KSS or SSS)—is monitored throughout the day, analyses sometimes tend to compute group-, day-, or condition-based averages and put aside individual temporal dynamics [41,42]. Yet, similar to the study by Buysse and colleagues [43] or the study by Schneider and colleagues [44], the present data show inter- and intraindividual variations in the participants’ complaints throughout the day. As such, relevant signs and symptoms of daytime complaints might not be captured by one-off measurements with a relatively long look-back period or may be lost when measured repeatedly but averaged across assessments, potentially including less affected moments. 

### 3.3. Strengths, Limitations, and Future Research

While our research raises important points for clinical and research views, it is crucial to reflect on future investigations. Both the study of Waldman et al. [40] and ours highlight the importance of considering the various terms used by patients and the consequences that daytime complaints related to sleep disorders can have. The current study focused on a patient population affected by obstructive sleep apnea or narcolepsy: two clinical populations known to experience daytime sleepiness but presenting different patterns and levels of symptoms and generally from quite different demographics (particularly in terms of age). We, therefore, argue that the terminology and descriptions, as well as the burden, of the daytime complaints are not diagnosis-specific but could instead relate to a wide group of patients suffering from daytime symptoms of sleep disorders or sleep disruptions. Future research endeavors should investigate daytime complaints amongst an enriched patient pool to hone reliable metrics and terminologies and to investigate whether and to what extent our observations are generalizable to other patient groups. In turn, it would be relevant to explore the overlap and distinctions between patients’ descriptions of daytime struggles and their scores on various scales. 

We performed this study amongst Dutch-speaking patients, and, although our findings align with articles and conceptualizations from English- and French-speaking countries, some differences might be due to the language. Indeed, while drowsiness is an important term in the concept of EDS according to Lopez et al. [11] and Martin et al. [18,19], our participants did not use this term nor any adjective associated with it. 

## 4. Conclusions

Our results highlight the richness of patients’ descriptions and the severity of struggles related to daytime sleepiness. Apart from insights into the terminology used by patients, the interviews shine light on the complexity of patients’ struggles. The images offered by the metaphors and consequences highlight the multidimensional effect that sleepiness, tiredness, fatigue, or exhaustion can have on daily lives. This effect seems even more harrowing by the persistent, repetitive presence of these daily experiences, even when we observe within-day variations. 

Our observations reinforce the importance of considering daytime complaints in patients’ experiences for more efficient diagnosis and treatment. We argue for a more extensive clinical interview of patients who suffer from (residual) daytime sleepiness or other daytime complaints and for a reconsideration or update of common scales or the combination of different scales that may jointly help guide the assessment of daytime complaints and the trajectory of treatment or intervention. These should not singularly target sleepiness but also assess concepts such as tiredness, fatigue, and exhaustion. Second, we recommend the use of various antipodes and levels of magnitude, particularly towards the more severe side of symptoms. Third, we would advise to also probe the consequences of EDS on cognition and mood. Last, explicit attention could be given to fluctuations in symptoms over time. To echo the advice formulated by Martin and colleagues [18], by diversifying diagnostical methods and by using measures of previously contrasted states (sleepiness, fatigue, exhaustion), researchers and clinicians might succeed in encapsulating the multidimensional burden of sleep disorders on patients’ quality of life. 

## 5. Materials and Methods

### 5.1. Study Design

This study adopted a qualitative approach to evaluate the daytime complaints experienced by patients with sleep disorders and how these symptoms vary as a function of time. The data were collected through individual semi-structured interviews conducted in Dutch, as this provided the opportunity to obtain a description of patients’ experiences in their own words and in-depth. The interviews were performed between the 5th of May and the 28th of June 2021.

### 5.2. Participants

The participants were recruited through convenience sampling via an open invitation distributed among Dutch sleep disorder associations. People could be included if they were diagnosed with a sleep disorder and spoke fluent Dutch. Because of the exploratory nature of this study, the sample size was set according to the achievement of data saturation (as defined by Guest et al. [45]). A total of twenty individuals participated in this qualitative study, of which eight were male and twelve female, with an age range between 20 and 75 years old (Median = 54.5, IQR = 13). The participants had received a diagnosis of either obstructive sleep apnea (N = 15) or narcolepsy (N = 5). A description of the diagnostical characteristics as made available by the participants can be found in Table 1.

### 5.3. Procedure

Due to COVID-19 regulations, the interviews were conducted via an online platform (Teams, Microsoft, Redmond, WA, USA, version 1.5.00.9163), like other qualitative studies at the time [46,47,48]. Therefore, people could only participate if they had access to a laptop or desktop with a camera. Before the start of the study, the participants were given the opportunity to ask questions or interrupt the interview. 

The semi-structured interview started with a short question pertaining to their sleep disorder diagnosis before focusing on the different related complaints they experienced during the day. From this point on and for the rest of the interview, care was taken to only use terms introduced by the participants to describe their experience so as not to bias them by introducing words or phrasings from the interviewers. Considering daytime symptoms, attention was paid to the physical, behavioral, and cognitive aspects. Afterwards, the dynamics of the daytime symptoms were discussed through questions regarding possible variation in the symptoms within and between days and their influencing factors. Last, the participants’ strategies or coping mechanisms used to alleviate their daytime symptoms were discussed. For demographic purposes, the age of the participants was asked, and their gender was noted. Once the main discussion points had been addressed and no new element had arisen, the participants were once again offered the opportunity to ask questions before the interview was concluded. The participants received monetary compensation.

### 5.4. Analysis

Each interview underwent a verbatim transcription, and the transcripts were analyzed with the MAXQDA 2022 software (version 22.1.0, VERBI GmbH, Berlin, Germany). The transcripts were divided into two groups corresponding to the two diagnoses and then reviewed and analyzed chronologically. Altogether, the content of the transcripts was processed and analyzed according to the steps described in the manual written by Saldana [49]. More specifically, the transcripts first underwent repetitive reading by V.T.R.V. and L.R. Once a good familiarity with the content had been achieved, V.T.R.V. and L.R. individually defined initial codes based on the methods of attribute coding (descriptive of the patients characteristics, e.g., diagnosis, treatment, list of daytime complaints), structural coding (labels guided by concepts relevant to the aim of the study, e.g., sleepiness, fatigue, time variation, symptoms, or signs), descriptive coding (inventory of the daytime complaints, their descriptors and antipodes, etc.), and subcoding (detailing initial bigger codes). With this first mix of coding methods, V.T.R.V. and L.R. compared and discussed codes in order to reach an agreement and finalize the first cycle of analysis. At this stage, the codes simply mirrored the structure of the interviews, the order, and the content of the questions. 

A second cycle of analysis added codes by means of using the versus coding (highlighting oppositions in patients’ descriptions) and axial coding (assembling or linking categories of codes) methods. The codes were created and added to the coding scheme, while taking care to re-evaluate prior transcripts with each new emerging code. While the terms corresponding to symptoms (common in the literature) were not always used verbatim, the meanings of the words or turns of phrase were analyzed and contextualized within the scientific literature to attempt a distinction of the symptoms. The codes related to daytime concerns not associated with sleepiness, tiredness, fatigue, or exhaustion were put aside as well as the interpretative codes on the attitudes and beliefs of the participants regarding their daytime experiences.

The entire coding scheme was then refined by V.T.R.V.: the codes were renamed, redefined, merged, or put aside if redundant. The resulting codes were organized into themes, meaning that the codes were united into a self-standing and self-explanatory construct. This process is described by Saldana under “Themeing the data” [49]. Together, the themes reflect the content of all the interviews (including interindividual similarities and differences) and the relation to the research aims. The themes were reviewed and discussed by V.T.R.V., K.C.H.J.S., Y.A.W.d.K., G.P., and S.O. 

## Figures and Tables

**Table 1:** Diagnostical characteristics of the participants.

Participant Code ^♦^	Gender	Age	Sleep Disorder(s) ^♦♦^	Treatment ^♦♦^
N1F21	Female	21	Narcolepsy	NM
N2F47	Female	47	Narcolepsy	Modafinil
N3M20	Male	20	Narcolepsy	Long-acting methylphenidate
N4M22	Male	22	Narcolepsy	Hydroxybutyric acid
N5F53	Female	53	Narcolepsy	Methylphenidate
A1M56	Male	56	Obstructive sleep apnea	CPAP
A2F54	Female	54	Obstructive sleep apnea Delayed sleep phase syndrome	CPAP
A3F55	Female	55	Obstructive sleep apnea Insomnia	Antidepressant Antipsychotic CPAP
A4F73	Female	73	Obstructive sleep apnea Insomnia	CPAP Mirtazapine
A5M50	Male	50	Obstructive sleep apnea	BPAP
A6F66	Female	66	Obstructive sleep apnea Chronic insomnia Restless leg syndrome	BPAP
A7M52	Male	52	Obstructive sleep apnea Central sleep apnea	APAP
A8F55	Female	55	Obstructive sleep apnea	CPAP
A9M49	Male	49	Obstructive sleep apnea	CPAP Sleep medication
A10F52	Female	52	Obstructive sleep apnea	CPAP
A11F62	Female	62	Obstructive sleep apnea Periodic limb movement disorder	CPAP
A12M69	Male	69	Obstructive sleep apnea	CPAP
A13F74	Female	74	Obstructive sleep apnea	CPAP
A14M61	Male	61	Obstructive sleep apnea	CPAP
A15F65	Female	65	Obstructive sleep apnea	CPAP

Note. NM = Not mentioned; CPAP = continuous positive airway pressure; BPAP = bilevel positive airway pressure; APAP = automatic positive airway pressure. ^♦^ The participant codes refer to the narcolepsy (N) or sleep apnea (A) diagnoses, the participant number, followed by the participant’s gender and age. ^♦♦^ This information was shared by the participants and does not constitute an exhaustive overview of clinical factors.

## Data Availability

The data underlying this article will be shared upon reasonable request to the corresponding author.
